# SRXN1 stimulates hepatocellular carcinoma tumorigenesis and metastasis through modulating ROS/p65/BTG2 signalling

**DOI:** 10.1111/jcmm.15693

**Published:** 2020-08-03

**Authors:** Xiufang Lv, Hailing Yu, Qianqian Zhang, Quanyong Huang, Xiaopeng Hong, Ting Yu, Huimin Lan, Chaoming Mei, Wenkai Zhang, Hui Luo, Pengfei Pang, Hong Shan

**Affiliations:** ^1^ Guangdong Provincial Key Laboratory of Biomedical Imaging and Guangdong Provincial Engineering Research Center of Molecular Imaging The Fifth Affiliated Hospital Sun Yat‐sen University Zhuhai China; ^2^ Department of Ultrasound The Fifth Affiliated Hospital Sun Yat‐sen University Zhuhai China; ^3^ Department of Hepatobiliary Surgery The Fifth Affiliated Hospital Sun Yat‐sen University Zhuhai China; ^4^ Center for Interventional Medicine The Fifth Affiliated Hospital Sun Yat‐sen University Zhuhai China

**Keywords:** BTG2, hepatocellular carcinoma, metastasis, SRXN1

## Abstract

Sulfiredoxin 1 (SRXN1) is a pivotal regulator of the antioxidant response in eukaryotic cells. However, the role of SRXN1 in hepatocellular carcinoma (HCC) is far from clear. The present study aims to elucidate whether SRXN1 participates in tumorigenesis and metastasis of HCC and to determine the molecular mechanisms. We found that SRXN1 expression was up‐regulated in HCC tissue samples and correlated with poor prognosis in HCC patients. We also observed that SRXN1 knockdown by transient siRNA transfection inhibited HCC cell proliferation, migration and invasion. Overexpression of SRXN1 increased HCC cell migration and invasion. B‐cell translocation gene 2 (BTG2) was identified as a downstream target of SRXN1. Mechanistic studies revealed that SRXN1‐depleted reactive oxygen species (ROS) modulated migration and invasion of HCC cells. In addition, the ROS/p65/BTG2 signalling hub was found to regulate the epithelial‐mesenchymal transition (EMT), which mediates the pro‐metastasis role of SRXN1 in HCC cells. In vivo experiments showed SRXN1 promotes HCC tumour growth and metastasis in mouse subcutaneous xenograft and metastasis models. Collectively, our results revealed a novel pro‐tumorigenic and pro‐metastatic function of SRXN1 in HCC. These findings demonstrate a rationale to exploit SRXN1 as a therapeutic target effectively preventing metastasis of HCC.

## 1 INTRODUCTION

Liver cancer ranks fifth among malignant cancers worldwide.^1^, ^2^ The incidence rate and mortality of liver cancer are higher in China than in any other country worldwide.^3^ HCC is the predominant type of primary liver cancer.^4^ Despite considerable advances in the diagnosis and therapy of HCC, the prognosis of most of patients is poor with a dismal five‐year survival rate of approximately 10%.^5^, ^6^ Both intrahepatic and extrahepatic metastases lead to relapse of these patients. It is imperative to identify novel molecular targets and determine their regulatory networks to curb metastasis of HCC. Studies exploiting these targets would benefit patients greatly by extending survival and improving patients’ quality of life.

Oxidative stress has been implicated in many physiological and pathological processes.^7^, ^8^ Disruption of the oxidative homeostasis of cells has been shown to be associated with HCC occurrence,^9^, ^10^ but the molecular mechanisms through which this occurs are not clarified.^10^, ^11^, ^12^ SRXN1 (sulfiredoxin 1) was discovered as an antioxidant protein. It catalyses the reduction of hyperoxidized peroxiredoxins to the reduced form, to thereby restoring their peroxidase activity.^13^, ^14^, ^15^ Several studies have shown the pro‐survival and pro‐metastasis function of SRXN1 in a wide array of malignant cancers.^16^, ^17^, ^18^, ^19^ However, the role of SRXN1 in HCC is far from clear. In this study, we identified the functions of SRXN1 in HCC progression and metastasis. Furthermore, we observed that inhibition of SRXN1 increased the level of reactive oxygen species (ROS) in cells, which modulated SRXN1 and its associated signalling pathway. The oxidative stress defence mechanism is of particular importance in tumour cell survival and proliferation. ROS levels in tumours are dysregulated not only due to tumour cells but also cellular components that make up the tumour microenvironment. However, excessive ROS induce preferential death of tumour cells. To curb redundant ROS, tumours utilize an antioxidant system to counteract oxidative pressure. Thus, targeting SRXN1 might be a useful strategy for selective killing of HCC cells.

B‐cell translocation gene 2 (BTG2) was originally identified as a member of the anti‐proliferative gene family.^20^, ^21^ BTG2 has been implicated in various cellular processes including cell proliferation, DNA repair, transcriptional regulation. There is evidence that BTG2 may be a target gene of p53.^22^, ^23^ Multiple regulators, including SET1DA and NF‐κB, modulate BTG2 expression.^24^, ^25^ However, the role of BTG2 in metastasis of malignant cancers has not been fully elucidated. Here in this study, we determined BTG2 as a downstream target of aberrantly up‐regulated SRXN1. EMT was characterized by transition from epithelial to mesenchymal state, through which the invasive potential of cells was enhanced. EMT was involved in progression and metastasis of HCC. In this study, we aim to explore the effect of BTG2 on EMT by detecting the regulation of BTG2 on EMT‐associated transcription factors. Determination of the role of BTG2 in HCC progression and metastasis unveil its novel functions in the progression and metastasis of malignant cancers.

The study was designed to explore the role of SRXN1 in the proliferation and metastasis of HCC. We aimed to investigate the expression of SRXN1 in HCC tumours and to elucidate how its aberrant expression modulates cellular signalling pathways in tumours. In a series of experiments, we found that SRXN1 expression was up‐regulated in HCC tumour tissue samples and determined the correlation between SRXN1 expression and HCC patient survival. SRXN1 was found to inhibit the expression of BTG2 by regulating ROS/p65 signalling. We also corroborated that EMT is involved in BTG2‐regulated tumour metastasis. Our study revealed a novel function of SRXN1 in HCC. These findings suggest that SRXN1 might be exploited as a therapeutic target in HCC.

## 2 MATERIALS AND METHODS

### 2.1 Human tumour specimens

A 102 pairs of HCC samples including tumour and adjacent morphologically normal tissues were collected from patients undergoing hepatectomy at The Fifth Affiliated Hospital of Sun Yat‐sen University between 2010 and 2017. Written consent was obtained from every patient prior to the experiment. Tissues were snap‐frozen immediately in liquid nitrogen and stored at −80°C until use. None of the patients in this study received chemotherapy, radiotherapy or immunotherapy before the operation. All experiments related to these tissue samples were approved by The Human Ethics Committee of The Fifth Affiliated Hospital of Sun Yat‐sen University in accordance with the World Medical Association Declaration of Helsinki.

### 2.2 Cell lines and cell culture

Hep3B and MHCC‐97H cells were obtained from Zhong Qiao Xin Zhou Biotechnology Co., Ltd. (Shanghai, China) and cultured in Dulbecco's modified Eagle's Medium (DMEM; Gibco, Big Cabin, UK) supplemented with 10% foetal bovine serum, 100 IU/mL penicillin and 100 μg/mL streptomycin. Cells were maintained in a humidified incubator at 37°C and 5% CO_2_.

### 2.3 Cell proliferation assay

Cell proliferation was measured using a label‐free, noninvasive cellular confluence assay with the IncuCyte Live‐Cell Imaging System (Essen Bioscience, Ann Arbor, MI, USA). Hep3B (5000 cells per well) or MHCC‐97H cells (6000 cells per well) were seeded overnight to allow adherence and entry into the logarithmic‐growth phase. The cells were placed in an incubator and imaged using a 10 × objective. The IncuCyte Analyzer was used to analyse real‐time cellular confluence data based on acquired images. Cell proliferation is expressed as the increase in the percentage of confluence.

### 2.4 Clonogenic assay

Briefly, cells were transfected with siRNAs or plasmids to manipulate SRXN1 expression. The transfected cells were seeded in six‐well plates at a density of 500 cells per well and allowed to grow for approximately 10‐14 days. Colonies were visualized after crystal violet staining.

### 2.5 Migration and invasion assays

Cells were seeded in 8‐μm‐pore inserts in transwell chambers (Corning, Corning, NY, USA) for migration and invasion assays. For the migration assay, 5 × 10^4^ cells and the respective control cells were plated in uncoated inserts and incubated for 24 hours in serum‐free medium. For the invasion assay, the inserts were coated with Matrigel (BD Biosciences, NJ, USA), and 1 × 10^5^ cells were plated in the serum‐free medium described above and cultured for 24 hours. Then 600 µL of culture medium containing 20% FBS was added to the lower chamber. The cells that had not migrated to the lower chamber were removed, and the cells attached to the bottom of the membrane were fixed with 4% paraformaldehyde, and stained with 5% crystal violet (Sigma‐Aldrich, Missouri, USA). Cells were then counted at 20 × magnification. The assays were repeated in three independent experiments.

### 2.6 RNA isolation and quantitative PCR (qPCR)

RNA extraction and real‐time qPCR were performed. Total RNA was extracted from cells and frozen tissue samples using the Total RNA Kit I (Omega, Norcross, GA, USA). Reverse transcription was accomplished using PrimeScript RT reagent kit with gDNA Eraser (TaKaRa, Kusatsu, Japan). For RT‐PCR, cDNA was amplified using TB Green Premix Ex Taq II (TaKaRa). RT‐PCR was performed by amplification of the target genes and GAPDH, which was used as a reference gene, using the QuantStudio 7 Flex Real‐Time PCR System (Thermo Fisher Scientific, Waltham, MA, USA). The primers used in RT‐PCR of the target and reference genes are as follows: SRXN1: 5′‐CAGGGAGGTGACTACTTCTACTC‐3′ and 5′‐CAGGTACACCCTTAGGTCTG A‐3′; BTG2: 5′‐AGGGTAACGCTGTCTTGTGG‐3′ and 5′‐TTGTAGTGCTCTGTGA GTGCC‐3′. GAPDH: 5′‐GCAGGGGGGAGCCAAAAGGGT‐3′ and 5′‐TGGGTGGCAGTGATGG CATCG‐3′.

### 2.7 Analysis of data from public cohorts

Data on the mRNA expression of SRXN1 in public cohorts including The Cancer Genome Atlas (TCGA) and GEO dataset GSE36376 were obtained for use in this study. Data on SRXN1 mRNA expression in 50 peritumor and 372 tumour tissues were extracted from the c‐BioPortal (http://www.cbioportal.org/) by downloading Liver Hepatocellular Carcinoma (LIHC) RNA‐seq data of TCGA (https://www.cancer.gov/aboutnci/organization/ccg/research/structural‐genomics/tcga/studied‐cancers/liver). Data on the mRNA expression of SRXN1 in 193 peritumor and 240 tumour tissues from GSE36376 were acquired from GEO bank on the NCBI website (https://www.ncbi.nlm.nih.gov/).

### 2.8 RNA interference and transfection

SRXN1 expression was knocked down by transiently transfecting MHCC‐97H and Hep3B cells with two SRXN1‐specific siRNAs chosen among four tested sequences (GenePharma, Shanghai, China). The sequences used in the knockdown experiments were as follows: 5′‐GCCGGCUCCAAAUUCCCAATT‐3′ and 5′‐GGACUACAUUCAGCUGCAATT‐3′. Briefly, siRNAs were transfected into the cells using Lipofectamine 2000 for 48 hours; the cells were subsequently cultured for use in experiments. Total RNA was prepared from cell lysates and subjected to PCR and Western blot analyses.

### 2.9 Western blot analysis

Briefly, cell pellets were prepared in RIPA buffer (Beyotime, Haimen, Jiangsu, China) containing a protease inhibitor cocktail. Nuclear and cytoplasmic proteins were fractionated using NE‐PER nuclear and cytoplasmic extraction reagents (Thermo Fisher Scientific) according to the protocol provided by the manufacturer. The lysates were separated by SDS‐PAGE and transferred to PVDF membranes. The membranes were immunoblotted with the indicated antibodies. Bands were visualized with ECL and imaged with ChemiDoc XRS (Bio‐Rad, CA, USA).

### 2.10 Immunofluorescence

Hep3B and MHCC‐97H cells were fixed with 4% formaldehyde in PBS for 5 minutes at room temperature and blocked with 1% BSA for 30 minutes. The cells were incubated overnight at 4°C with an anti‐Snail antibody (1:200, Abcam, Cambridge, UK), an anti‐E‐cadherin antibody (1:100, Cell Signaling Technology, Boston, MA, USA), or an anti‐p65 antibody (1:100, Cell Signaling Technology) and then incubated with Alexa Fluor 488‐labelled and Alexa Fluor 594‐labelled secondary antibodies (1:200, Invitrogen, Carlsbad, CA, USA) for 1 hour at room temperature. Each step was followed by three 5‐minute washes in PBS. The prepared specimens were counterstained with a fluorescence‐antiquenching solution containing DAPI (Solarbio, Beijing, China) for 10 minutes and observed with a confocal microscope (Zeiss, Jena, Germany).

### 2.11 Immunohistochemistry (IHC)

Tissue slices were incubated in xylene for deparaffinisation and in decreasing concentrations of ethanol for rehydration. Subsequently, 3% hydrogen peroxide solution was applied to quench endogenous peroxidase activity. Antigen retrieval was achieved using high‐pressure methods. After blocking of the slices with goat serum, the slices were incubated with the following primary antibodies overnight at 4℃: anti‐SRXN1 (1:50, Proteintech, Illinois, IL, USA), anti‐Ki67 (1:200, Cell Signaling Technology), and anti‐BTG2 (1:200, Abcam). A secondary antibody from a horseradish peroxidase‐polymer anti‐mouse/rabbit IHC kit (Zhongshan Golden Bridge, Beijing, China) was added and the samples were incubated for 1 hour at room temperature. The samples were then developed in diaminobenzidine (DAB) reagent, counterstained with haematoxylin and mounted with permount.

### 2.12 ROS measurement

ROS were measured using CellROX^®^ Deep Red Reagent (Life Technologies, Grand Island, NY, USA) according to the manufacturer's protocol. In brief, Hep3B and MHCC‐97H cells were transfected with SRXN1‐siRNAs. After 72 hours, CellROX^®^ deep red reagent was added to medium at 1:500 in the dark for 30 minutes at 37℃, The medium was then removed and the cells were washed three times with PBS, collected and resuspended in 500 μL PBS for flow cytometry acquisition.

### 2.13 Luciferase reporter assays

Snail‐Gluc, Vimentin‐Gluc, E‐cadherin‐Gluc, and GAPDH‐Gluc plasmids were purchased from GeneCopoeia (Guangzhou, Guangdong, China). All plasmids contained SEAP signals. Cells were transfected with the indicated plasmids, and the Dual Luminescence Assay Kit (GeneCopoeia) was used to capture the signals in each well according to the manufacturer's instructions. The ratio of Gluc to SEAP was plotted to graph triplicate results.

### 2.14 Animal experiments

For in vivo experiments, Hep3B cells stably expressing SRXN1‐shRNA were constructed using lentiviral methods. Briefly, two shRNA sequence based on pLKO.1‐puro vectors targeting SRXN1 were synthesized: 5′‐CCGGCCCAGGGAGGTGACTACTTCTCTCGAGAGAAGTAGTCACCTCCCTGGGTTTTTG‐3′ and 5′‐CCGGCGTGCCGCTGAGCGTGCTCATCTCGAGATGAGCACGCTCAGCGGCACGTTTTTG‐3′. The knockdown efficiency was verified by Western blot assays. One shRNA with best knockdown efficiency was chosen and transfected into 293T cells together with packaging plasmids. The lentiviruses were then collected and transfected into Hep3B cells to construct stable cell lines. For the tumour growth assay, 5 × 10^6^ cells were subcutaneously inoculated into the left shoulders of 6‐week‐old male Balb/c nude mice. Beginning ten days after injection, tumour volume and bodyweight were recorded every 5 days. Tumour volume was calculated according to the formula length × width × height × π/6. Forty days after inoculation, the tumours were harvested and photographed. Tumour tissue samples were subjected to IHC using the indicated antibodies. For metastasis assays, 1 × 10^6^ cells were injected into mice via the tail vein. Fifty days after injection, the mice were killed, and the lungs were excised and subjected to haematoxylin and eosin (HE) staining. Metastatic HCC nodules were counted and statistically analysed. All animal experiments were approved by The Animal Ethics Committees of the Fifth Affiliated Hospital of Sun Yat‐sen University.

### 2.15 Statistical analysis

All figures and data are representative of at least three independent experiments. Data are presented as means ± standard deviation (SD). Statistical analysis was conducted using GraphPad Prism 7. Significant differences were evaluated using Student's *t test*, and *P* < 0.05 was considered statistically significant. Kaplan‐Meier survival and the log‐rank tests were used to analyse the correlation between SRXN1 expression and HCC patients’ survival.

## 3 RESULTS

### 3.1 SRXN1 is highly expressed in HCC, and its expression is clinically significant

**FIGURE 1:**
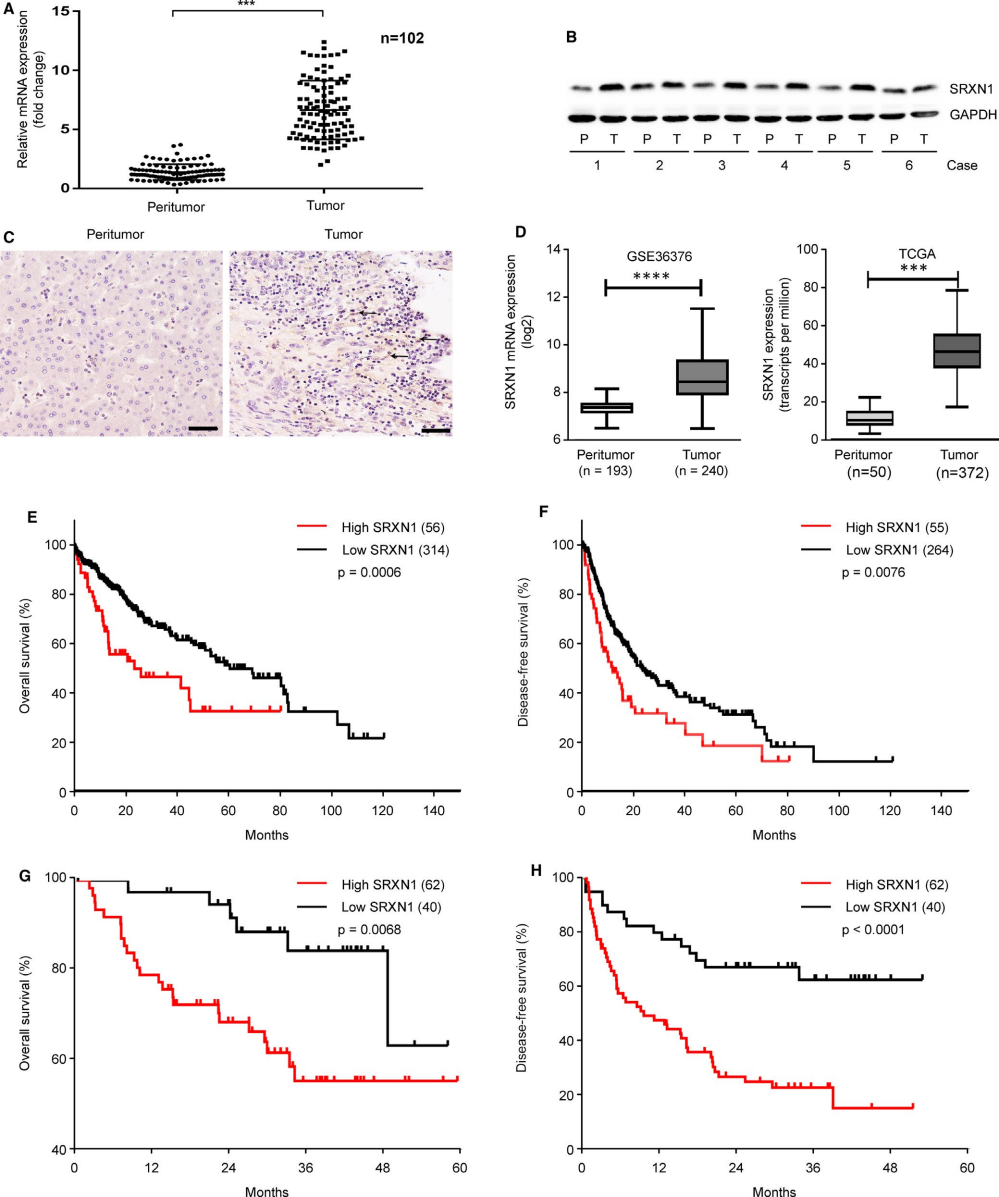
SRXN1 expression is up‐regulated in HCC tumour tissues and is associated with poor survival. A, qPCR analysis of SRXN1 mRNA expression in 102 pairs of HCC and adjacent control tissues. *** indicates *P* < 0.001. B, Western blot analysis of SRXN1 protein expression in 6 representative pairs of HCC tissue samples. P indicates peritumor tissue; T indicates tumour tissue. C, Representative IHC images (20 × magnification) are shown. The black arrows pointing to the brown puncta indicate positive staining for SRXN1 in the tumour sections. Scale bar: 50 μm. D, mRNA expression of SRXN1 reported in public databases. GSE36376 (left panel) and TCGA (right panel) datasets. *** indicates *P* < 0.001. E‐F, Analysis of overall survival and disease‐free survival analyses in HCC patients in the TCGA datasets stratified by SRXN1 mRNA expression. G‐H, Analysis of overall survival and disease‐free survival analyses in HCC patients in our own collected tissues. (n = 102)

We first examined the expression of SRXN1 in 102 pairs of previously collected human HCC tumour and peritumor tissue samples. Both the mRNA and protein expression of SRXN1 were elevated in the tumour tissue compared with the peritumoral tissue (Figure 1A,B). IHC staining of paraffin‐embedded HCC tissue samples also revealed more positive staining for SRXN1 in the tumour tissue than in the corresponding peritumoral tissue (Figure 1C). We validated our findings using data from public databases including TCGA and GSE cohorts. Consistent with our results, the SRXN1 mRNA levels reported in these databases were higher in tumour tissue samples than in their peritumoral counterparts (Figure 1D). Moreover, we found that high expression of SRXN1 was correlated with poor prognosis (low rates of overall survival and disease‐free survival) of HCC patients in both the TCGA data (Figure 1E,F) and our collected tissue samples (Figure 1G,H). These results indicate that SRXN1 was highly expressed in HCC tissue and that its expression is negatively correlated with patient survival.

### 3.2 SRXN1 expression is associated with the proliferation of HCC cells

**FIGURE 2:**
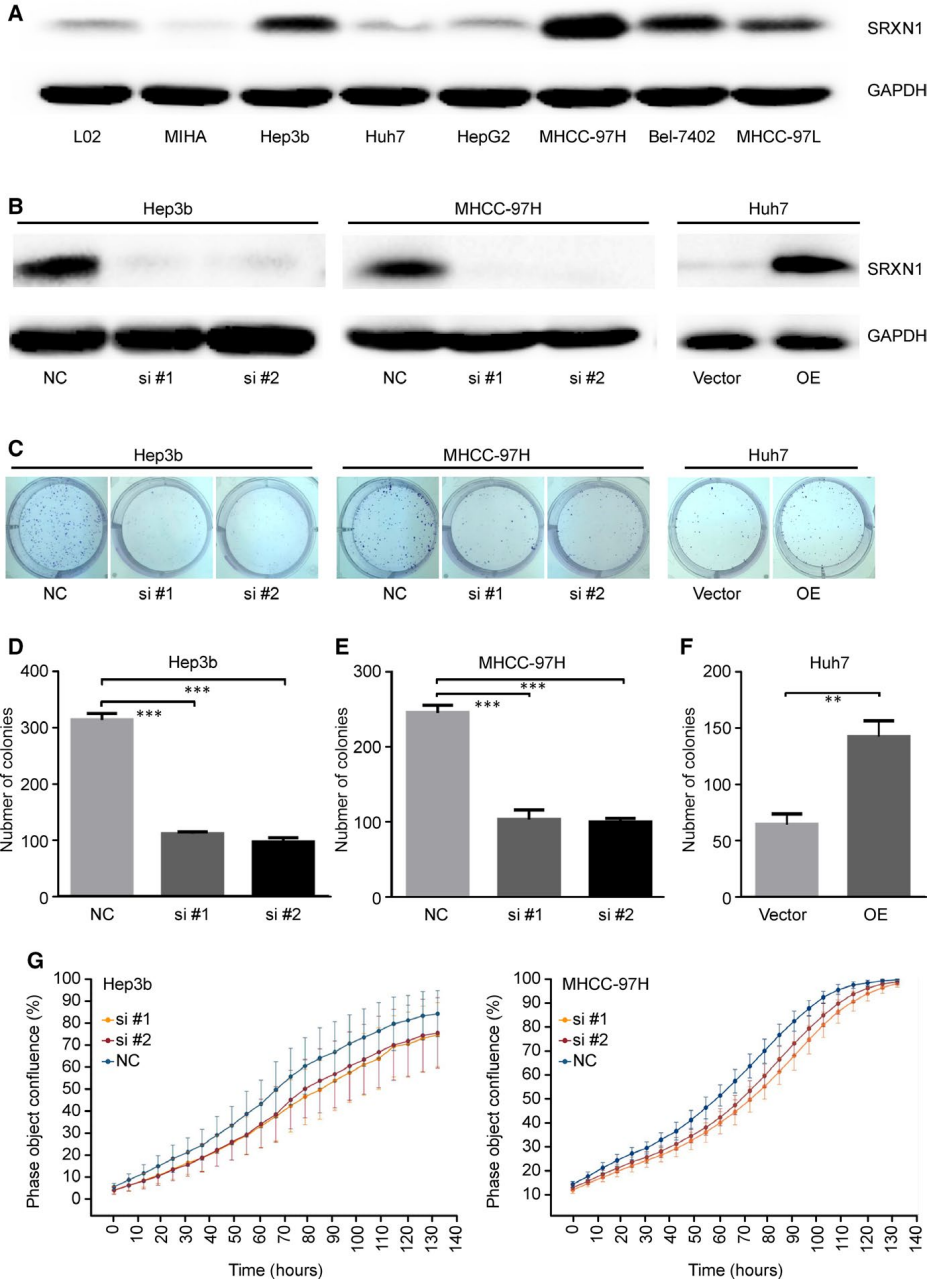
SRXN1 has a tumorigenic role in HCC cells. A, Differential expression of SRXN1 was detected in different HCC cell lines by Western blot analysis. GAPDH was used to normalize protein loading. B, The expression of SRXN1 in Hep3B and MHCC‐97H cells after siRNA‐mediated knockdown and in Huh7 cells transfected with either a control vector or SRXN1‐containing plasmids (OE) was detected by Western blot analysis. C, Colony formation assays were performed using cells treated with siRNA or with overexpression (OE) plasmids targeting SRXN1. D‐F, Statistical analysis of the results of three independent colony formation assays using Hep3B, MHCC‐97H and Huh7 cells. *** indicates *P* < 0.001; ** indicates *P* < 0.01. G, The extent of confluence of Hep3B and MHCC‐97H cells was recorded and plotted against time. Curves of different colours represent control and SRXN1‐knockdown cells

To clarify the function of SRXN1 in HCC, we first examined the expression of SRXN1 in various HCC cell lines. SRXN1 expression in Hep3B and MHCC‐97H cells was relatively high, while it was the lowest in Huh7 cells (Figure 2A). We screened two useful siRNAs sequences that successfully knocked down the expression of SRXN1 as revealed by Western blot assays. In addition, SRXN1 overexpression was achieved by transient transfection of Huh7 cell with a plasmid containing SRXN1 sequences (Figure 2B). A colony formation assay (CFA) was used to detect the tumorigenic ability of SRXN1. Compared with the control group, cells with SRXN1 knockdown formed fewer colonies, where SRXN1 overexpression augmented colony formation ability by Huh7 cells (Figure 2C). A statistical analysis of CFA assays presented Figure 2D‐F. Moreover, the proliferation of Hep3B and MHCC‐97H cells decreased markedly after SRXN1 knockdown (Figure 2G). These results collectively highlight a tumorigenic role for SRXN1 in HCC.

### 3.3 SRXN1 is required for EMT‐associated migration and invasion by HCC cells

**FIGURE 3:**
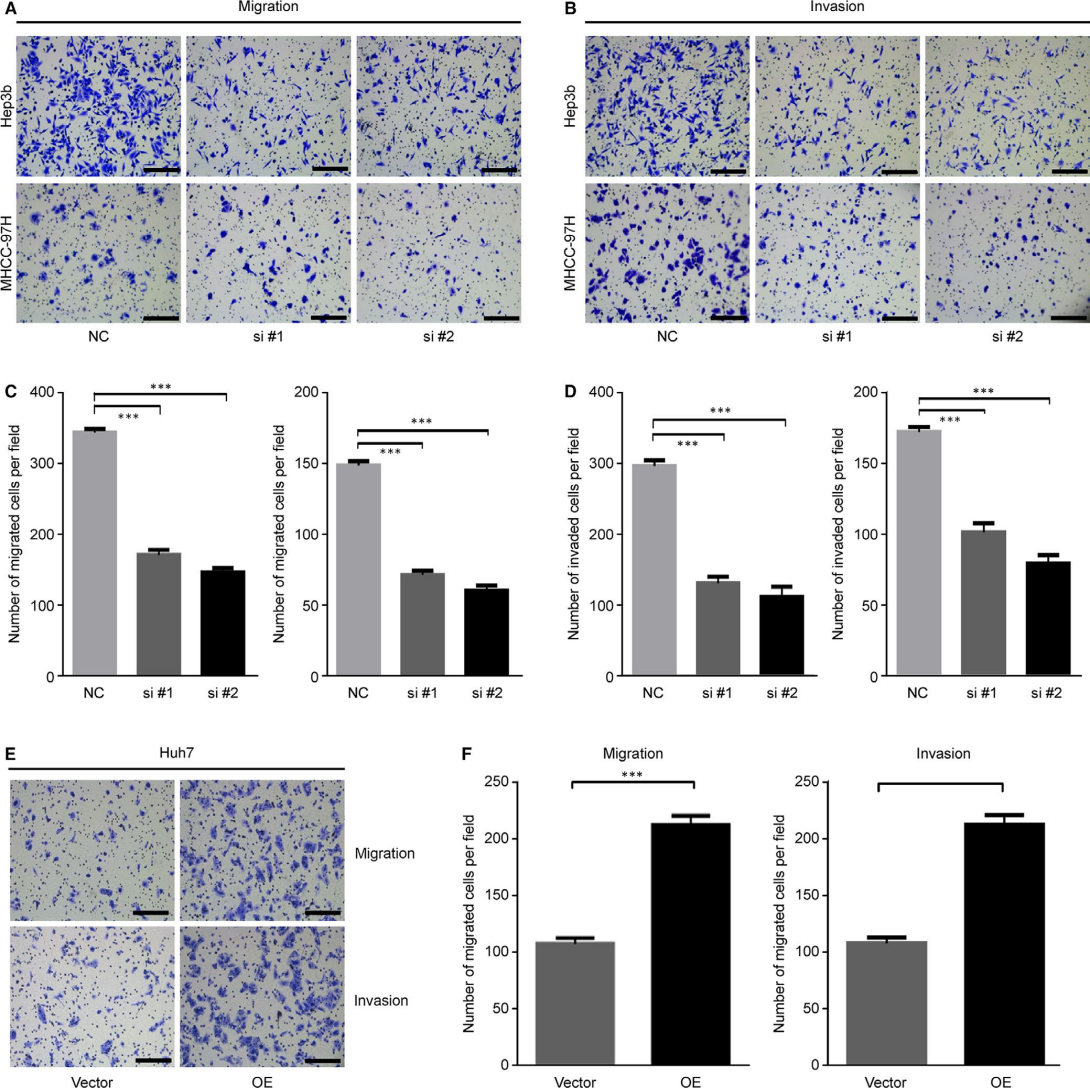
SRXN1 is involved in regulating migration and invasion by HCC cells. A, Transwell assay detecting migration by Hep3B (upper panel) and MHCC‐97H cells (lower panel) with knocked down SRXN1 expression. Scale bar: 100 μm. B, Transwell assay detecting invasion by Hep3B (left panel) and MHCC‐97H cells (right panel) with knocked down SRXN1 expression. Scale bar: 100 μm. C, Statistical analysis of migration of Hep3B (left panel) and MHCC‐97H cells (right panel). Five random fields of migrated cells were counted, and the results were subjected to t* test* analysis. *** indicates *P* < 0.001. D, Statistical analysis of invasion by Hep3B (left panel) and MHCC‐97H cells (right panel). Five random fields of invading cells were counted, and the results were subjected to t test analysis. *** indicates *P* < 0.001. E, Transwell assay detecting migration (upper panel) and invasion (lower panel) of SRXN1‐overexpressing Huh7 cells. V indicates vector, and OE indicates overexpression. F, Statistical analysis of migration (left panel) and invasion of Huh7 cells in two groups. *** indicates *P* < 0.001

To investigate whether SRXN1 is essential for migration and invasion by HCC cells, we knocked down SRXN1 expression using siRNAs. In a transwell assay, we observed that decreased expression of SRXN1 resulted in reduced numbers of migrating and invading Hep3B and MHCC‐97H cells (Figure 3A,B). Overexpression of SRXN1 in Huh7 cells increased the number of migrating and invading cells (Figure 3E). Statistical analysis indicated that modulation of SRXN1 expression caused changes in migration and invasion by HCC cells (Figure 3C,D,F). Collectively, these results show that SRXN1 plays a role in migration and invasion of HCC cells, implying a pro‐metastatic role of SRXN1.

**FIGURE 4:**
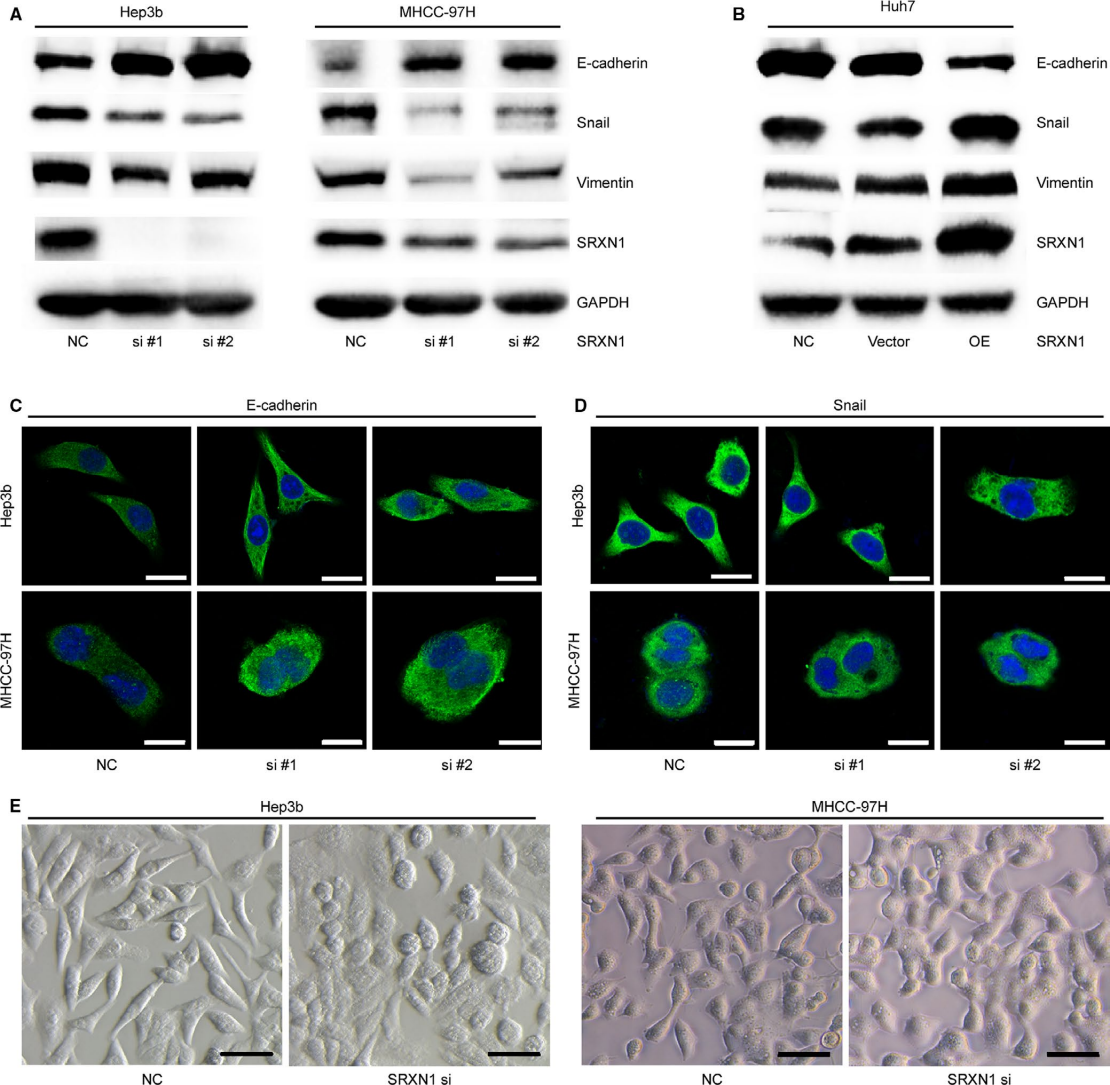
SRXN1 modulated EMT in HCC cells. A, Western blot analysis detecting the expression of EMT‐related molecules after SRXN1 knockdown in Hep3B cells (left panel) and MHCC‐97H cells (right panel). B, Western blot analysis detecting the expression of EMT‐related molecules after SRXN1 overexpression in Huh7 cells. GAPDH was used as an internal control. C, Immunofluorescence results for E‐cadherin expression and localisation in Hep3B and MHCC‐97H cells. Green signal: E‐cadherin; blue signal: nucleus. Cells were observed with a 63X oil lens. Scale bar: 20 μm. D, Immunofluorescence results for Snail expression and localisation in Hep3B and MHCC‐97H cells. The green signal represents Snail, and the blue signal indicates the nucleus. Scale bar: 20 μm. E, Morphology image acquired with a phase‐contrast microscope using a 10X lens. Hep3B (Left) and MHCC‐97H (right) cells were transfected with either a scrambled siRNA or siRNAs targeting SRXN1. Scale bar: 100 μm

EMT is a pivotal process that promotes migration and invasion. Thus, we measured the expression of EMT‐related molecules in HCC cells after SRXN1 knockdown or overexpression. As shown in Figure 4A, the expression of the epithelial marker E‐cadherin was marginally enhanced after SRXN1 knockdown, while the expression of the mesenchymal markers Snail and Vimentin was concordantly decreased with SRXN1 knockdown. Conversely, overexpression of SRXN1 enhanced the expression of mesenchymal markers while inhibiting the expression of epithelial markers (Figure 4B). Complementing these results, immunofluorescence experiments showed that E‐cadherin expression increased in the cytoplasm, while Snail expression decreased in the nucleus after SRXN1 knockdown (Figure 4C,D). Furthermore, we observed that SRXN1 knockdown suppressed the development of elongated cell morphology, indicating that the EMT was inhibited in the context of SRXN1 knockdown (Figure 4E). Based on these findings, we concluded that SRXN1 promotes the migration and invasion by HCC cells by modulating the EMT program.

### 3.4 BTG2 is a downstream target of SRXN1

**FIGURE 5:**
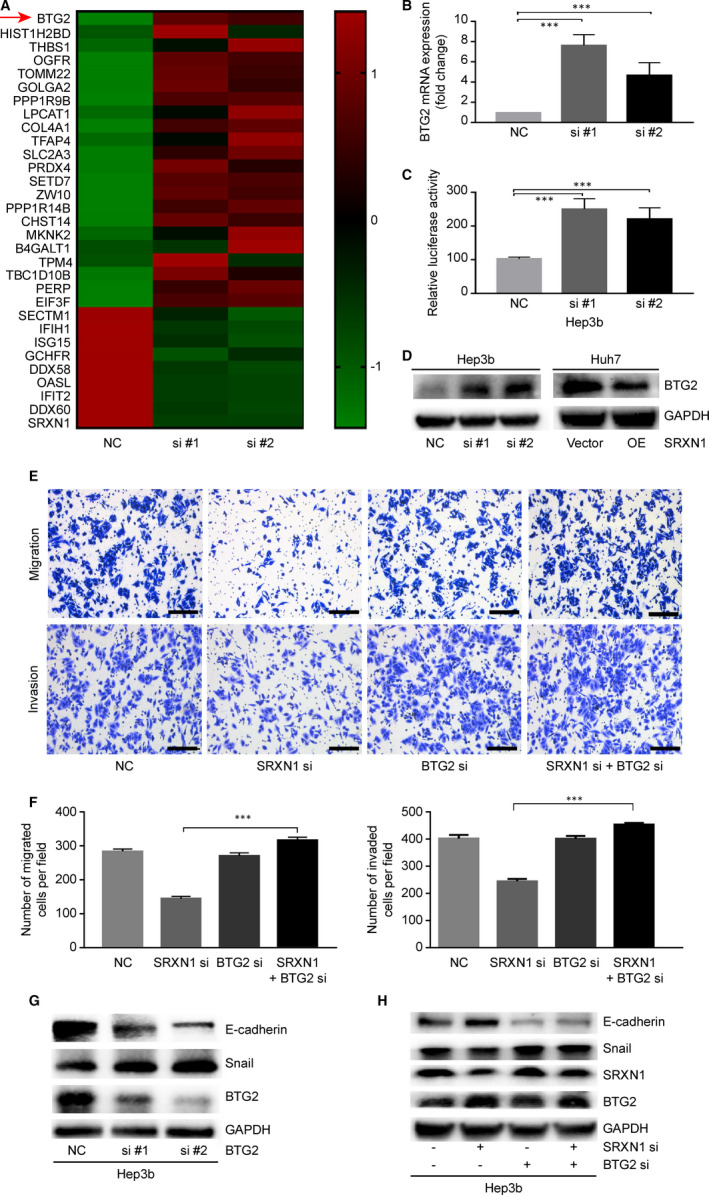
BTG2 is a downstream target of SRXN1. A, Heatmap of differentially expressed genes in control and SRXN1‐knockdown Hep3B cells. The scale bar indicates a log2‐fold change in gene expression. B, mRNA expression of BTG2 determined by qPCR. *** indicates *P* < 0.001. C, Dual‐luciferase assay detection of BTG2 promoter‐driven luciferase activity in control and SRXN1‐knockdown cells. #1 and #2 indicate two separate siRNAs sequences targeting SRXN1. *** indicates *P* < 0.001. D, Western blot assays detecting the expression of BTG2 after SRXN1 knockdown. E, Transwell assays of Hep3B cells analysing the migratory and invasive abilities of the cells after SRXN1 and BTG2 silencing. F, Statistical analysis of the migration and invasion of Hep3B cells in the context of SRXN1 and BTG2 silencing. The migrated or invaded cells in five random fields were counted, and the data were subjected to t test analysis. *** indicates *P* < 0.001. G, Effect of BTG2 knockdown on EMT‐related molecules. si #1 and si #2 represent two separate siRNAs sequences targeting BTG2. GAPDH was used to normalize the protein loading among different samples. H, Western blot assays detecting the expression of E‐cadherin, Snail, BTG2 and SRXN1 in Hep3B cells with SRXN1 silencing alone or in combination with BTG2 silencing

We concluded that SRXN1 is overexpressed in HCC and that its expression is clinically significant. Furthermore, we found that SRXN1 promotes proliferation, invasion and migration by HCC cells. These findings led us to explore how SRXN1 implements these biological functions. To address this issue, we conducted RNA‐seq experiments using control and SRXN1‐knockdown Hep3B cells. From the sequencing results (Figure 5A), we observed that BTG2 mRNA expression increased significantly after SRXN1 knockdown. We validated the mRNA expression results by qPCR analysis. We observed that SRXN1 mRNA expression in knockdown cells was much higher than that in control cells (Figure 5B). Additionally, we transfected Hep3B cells with the BTG2 promoter sequence labelled with luciferase. Relative luciferase activity was markedly enhanced when SRXN1 expression was suppressed (Figure 5C). We examined the protein level of BTG2 and discovered that BTG2 expression was elevated in Hep3B cells when SRXN1 expression was knocked down. The expression of BTG2 consistently decreased when SRXN1 was overexpressed in Huh7 cells (Figure 5D). To explore whether BTG2 is the critical factor in the mechanism of action of the function of SRXN1, we conducted a series of experiments. First, the inhibitory effects on migration and invasion induced by SRXN1 knockdown were reversed by BTG2 silencing (Figure 5E,F). Previously, we showed that SRXN1‐induced migration and invasion were associated with EMT. Thus, we postulated that BTG2 may regulate EMT downstream of SRXN1. The changes in protein expression observed in Western blot assays showed that BTG2 was successfully knocked down. The expression of EMT markers was also influenced by BTG2 knockdown (Figure 5G). Importantly, the SRXN1 silencing‐induced suppression of EMT was reversed by BTG2 silencing (Figure 5H). We concluded that BTG2 is a direct downstream target in SRXN1‐regulated metastasis of HCC.

### 3.5 ROS‐mediated p65 signalling participates in the regulation of BTG2 expression

**FIGURE 6:**
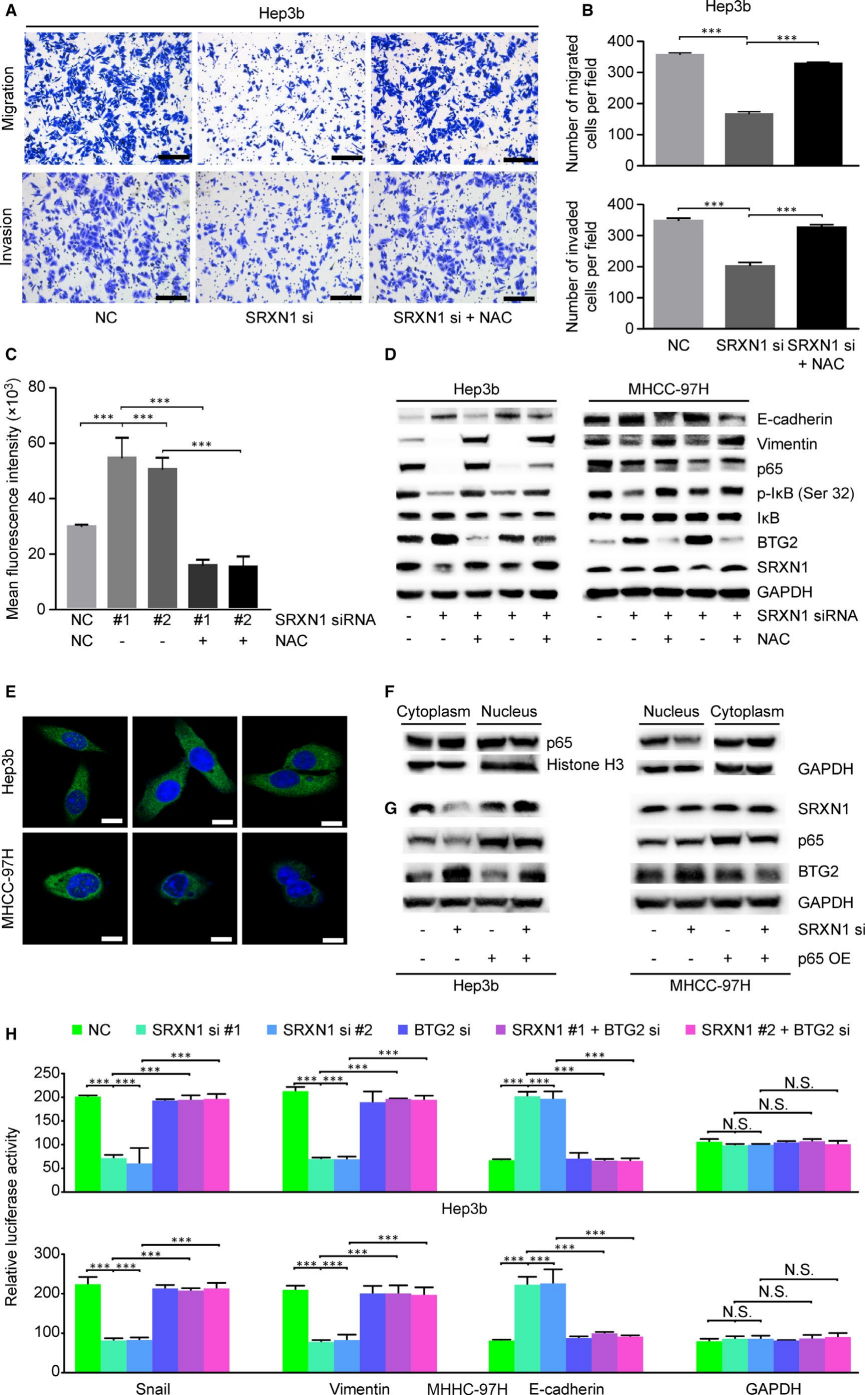
ROS/p65/BTG2 signalling mediates the pro‐tumorigenic activity of SRXN1. A, Transwell assay detecting the migration and invasion of SRXN1‐silenced HCC cells pretreated with or without NAC (5 μmol/L). B, Statistical analysis of cell counts in five random fields of migrated or invaded cells. *** indicates *P* < 0.001. C, ROS levels in Hep3B cells were monitored after treatment of the cells with or without SRXN1 siRNA. Mean fluorescence was recorded in three independent experiments. NAC was added 24 h before detection (columns 4 and 5). D, Western blot assays detecting the expression of EMT‐related and p65 signalling proteins. GAPDH was used to ensure equal loading among groups. E, Representative images showing immunostaining for p65. The nuclei were counterstained with DAPI solution. Green signals indicate p65, and blue signals indicate the nucleus. Scale bar represents 20 μm. F, Distribution of p65 after SRXN1 knockdown as detected by Western blot analysis. GAPDH was used as an internal control for cytoplasmic fractions, and histone H3 was used as an internal control for nuclear fractions. G, Western blot analysis of the expression of BTG2 in SRXN1‐knockdown cells after p65 overexpression. H, Luciferase detection of EMT‐related promoter activity. The GAPDH promoter was used as a control. The normalized luciferase activity was calculated from three independent experiments and plotted

SRXN1 was previously reported to regulate antioxidant responses in cancer cells.^26^ This finding led us to hypothesize that SRXN1 may regulate cellular ROS levels in a way that affects migration and invasion by HCC cells. To test our hypothesis, we examined the ROS levels in SRXN1‐knockdown cells. Figure 6C shows that ROS levels increased when SRXN1 was inhibited. Correspondingly, cells were transfected with a siRNA targeting SRXN1 and treated with NAC, a ROS scavenger. We found that the SRXN1 silencing‐induced inhibition of migration and invasion by Hep3B cells was abolished in cells that received NAC treatment (Figure 6A,B). p65 is a nuclear transcription factor that induces a plethora of cellular responses including antioxidant activity, metastasis and survival of tumour cells.^27^ We analysed the combined effect of SRXN1 silencing and NAC treatment on p65 signalling. As shown in Figure 6D, silencing of SRXN1 inhibited p65 signalling in HCC cells. I‐κb is a negative regulator of p65. Phosphorylation of I‐κb at Ser 32 causes its degradation, and this releases p65 into the nucleus to activate downstream targets.^28^ As shown in Figure 6D, we observed reduced phosphorylation of I‐κb at Ser 32 in cells in which SRXN1 expression was suppressed, implying that SRXN1 knockdown caused activation of I‐κb and thus suppressed p65. Shuttling of p65 from the cytoplasm to the nucleus is indispensable for its function. Therefore, we performed immunofluorescence experiments (Figure 6E). SRXN1 knockdown resulted in reduced p65 accumulation in the nucleus, as shown by a reduced frequency of green puncta (p65) in the nucleus. Additionally, by subcellular extraction of proteins from the nucleus and cytoplasm, we observed reduced p65 accumulation in the nucleus after SRXN1 knockdown (Figure 6F). Notably, overexpression of p65 (OE) dramatically inhibited the up‐regulation of BTG2 expression that was otherwise induced by SRXN1 silencing (Figure 6G). These results strongly indicate that SRXN1 modulates p65 signalling, leading to the inhibition of BTG2.

To determine whether ROS‐regulated p65 signalling modulates EMT and whether BTG2 is essential for EMT, we examined the expression of E‐cadherin and Vimentin. As shown in Figure 6D, the changes in E‐cadherin and Vimentin expression caused by SRXN1 silencing were reversed by NAC treatment. Correspondingly, SRXN1‐induced BTG2 up‐regulation was quenched by NAC treatment. Luciferase‐conjugated promoters of EMT‐related molecules were used to analyse the effect of BTG2 silencing on EMT. As shown in Figure 6H, the modulation of EMT markers by SRXN1 was reversed by BTG2 silencing. These results verify our hypothesis that SRXN1‐modulation of ROS/p65 signalling is the key mechanism through which SRXN1 regulates BTG2 expression and EMT and suggests that BTG2 is the pivotal factor that acts downstream of SRXN1 in the modulation of EMT.

### 3.6 SRXN1 is a critical factor in tumorigenesis and metastasis in vivo

**FIGURE 7:**
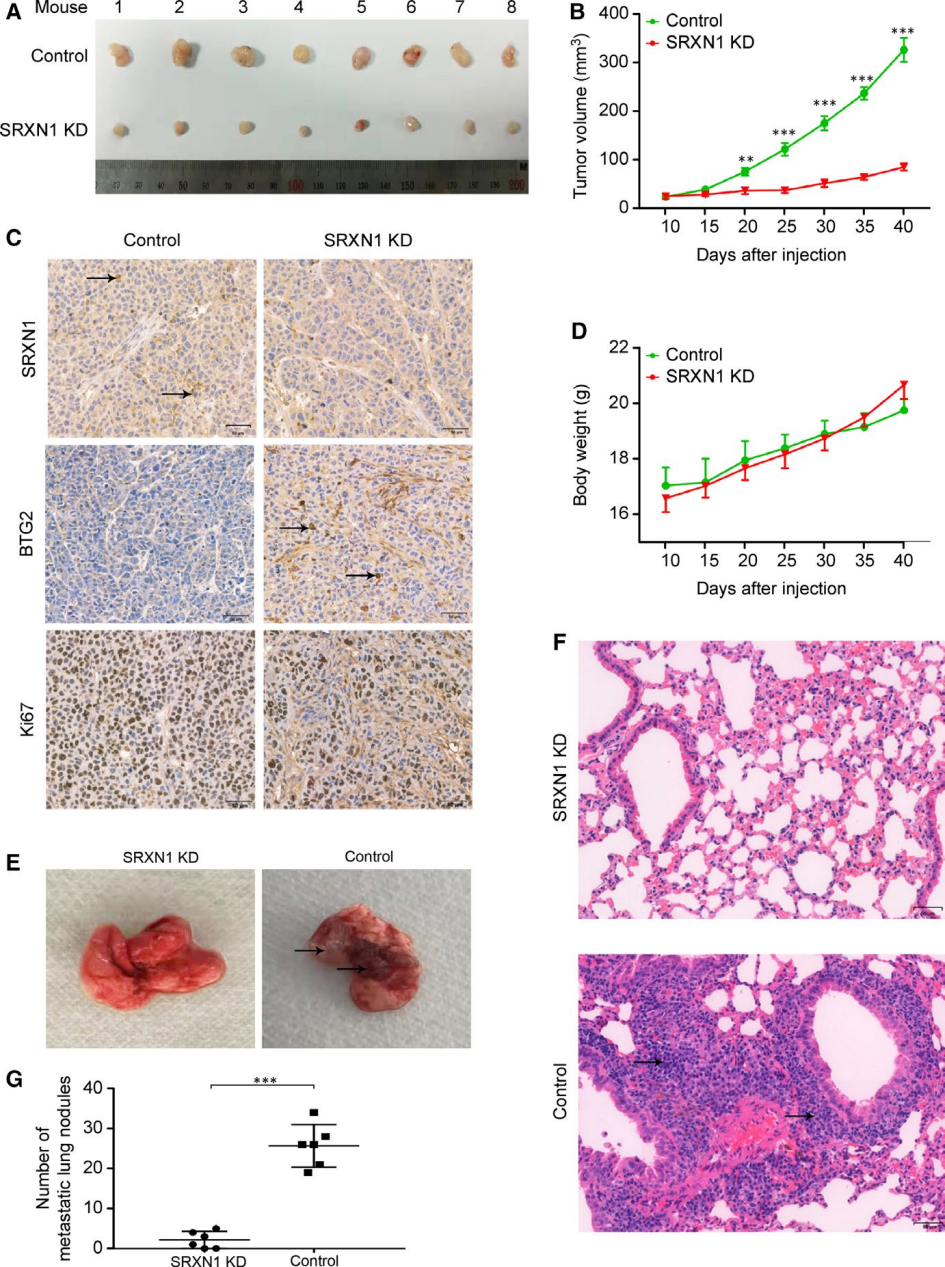
SRXN1 inhibits tumour growth and metastasis in vivo. A, Forty days after subcutaneous tumour cell inoculation, tumours were harvested and photographed (n = 8). B, The growth of tumours in the two groups was compared over time. Tumour volumes were calculated and recorded every 5 d. *** indicates *P* < 0.001; ** indicates *P* < 0.01. C, The bodyweight of each mouse was recorded every 5 d; the results are plotted against time. D, IHC analysis of tumour sections from the control and KD groups using antibodies against SRXN1, Ki67 and BTG2. The black arrow highlights the positive staining of related molecules in each panel. Scale bar: 50 μm. E, Lungs were harvested from mice in the control and KD groups 60 d after injection. Metastatic nodules were observed in the control group but not in the KD group. The black arrow indicates metastatic nodules in the lungs. F, HE staining of lung tissue samples from the control and KD groups. The black arrow indicates a metastatic nodule. Scale bar: 50 μm. G, Nodules in the lungs were counted and statistically analysed between control and KD groups. (n = 6)

The results of experiments described above show that SRXN1 promotes the migration, invasion and proliferation by HCC cells in vitro. To further clarify SRXN1’s role in HCC tumorigenesis, we conducted in vivo experiments in mouse xenograft models. We verified the expression of SRXN1 in Hep3B cells using previous two sequences. As shown in Figure S1, we determined sequence 1 for following animal experiments. Subsequently, we inoculated control or SRXN1‐knockdown (KD) cells into nude mice subcutaneously or via tail vein injection. As shown in Figure 7A, the tumours produced by KD cells were much smaller than those produced by control cells. The curves for tumour volume also showed that the tumours in the KD group grew much more slowly than those in control group (Figure 7B). Bodyweight analysis showed that KD cells displayed no toxicity in mice (Figure 7C). Immunohistochemical staining for Ki67 showed that SRXN1 knockdown suppressed the growth of cells in the tumour bulk. Additionally, SRXN1 expression was successfully knocked down in KD cells, as positive brown puncta were seldom observed in KD tumour tissue samples. Consistent with the in vitro results, BTG2 expression was enhanced in SRXN1‐knockdown tissue samples (Figure 7D).

In the metastasis model, we observed fewer nodules in the lungs of the mice in the KD group than in those of the mice in the control group (Figure 7E). HE staining also showed that metastatic nodules were present in the lungs of the mice in the control group and that fewer nodules were present in the lungs of the mice in the KD group (Figure 7F). The results obtained in six mice from each group were analysed statistically (Figure 7G), and it was concluded that SRXN1 knockdown dramatically inhibited HCC metastasis.

**FIGURE 8:**
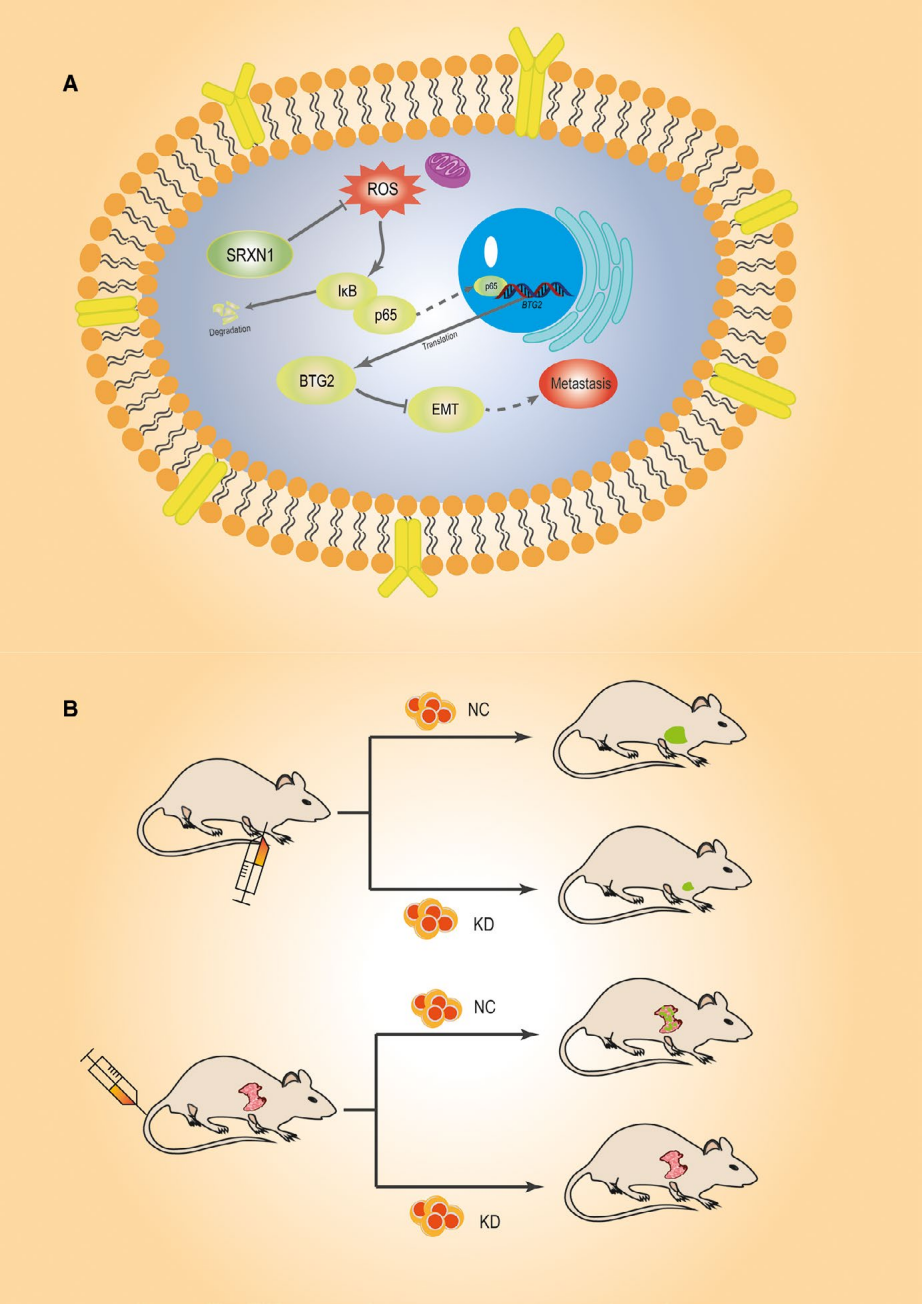
Schematic representation of the roles of SRXN1 in HCC tumorigenesis and metastasis. A, The SRXN1/p65/BTG2 signalling axis modulates cell growth and metastasis in vitro. SRXN1 constrains cellular ROS levels, which results in the degradation of I‐κb and the activation of p65. The accumulation of p65 in the nucleus suppresses the expression of BTG2 and ultimately promotes HCC cell growth and EMT. B, In vivo analysis of the role of SRXN1 in mouse models. Tumour growth was monitored in a subcutaneous xenograft model established with control or SRXN1‐silenced cells. Meanwhile, Hep3B cells were injected into mice via the tail vein. Metastatic nodules in the lungs of the mice were monitored and counted to analyse the pro‐metastatic role of SRXN1

Taken together, the results described above show that SRXN1 promotes proliferation, migration and invasion by HCC cells by affecting ROS‐mediated p65/BTG2 signalling. SRXN1 suppresses cellular ROS, resulting in the activation of p65 through enhancement of I‐κB degradation. Accumulation of p65 in the nucleus inhibits BTG2 expression. Suppression of BTG2 augments EMT, thereby inducing HCC cell migration and invasion (Figure 8A). Based on the results of in vivo experiments using stable SRXN1‐knockdown and control cells, we conclude that SRXN1 knockdown attenuates the growth and metastasis of HCC in the mouse model (Figure 8B).

## 4 DISCUSSION

SRXN1 was originally described as an antioxidant molecule involved in the maintenance of oxidative homeostasis.^29^ Aberrant oxidative regulation has been observed in various types of cancer cells.^30^, ^31^ Mounting evidence indicates that perturbation of oxidative homeostasis is involved in tumorigenesis.^32^, ^33^ However, the roles of antioxidant molecules in malignant cancers, especially liver cancer, are relatively unclear. ROS are pivotal mediators of oxidative stress and are involved in the regulation of survival of cancer cells.^34^ Considerable evidence indicates that ROS play multiple roles in cancer.^35^, ^36^, ^37^ On one hand, ROS are essential for survival of cancer cells and tumour growth. On the other hand, excessive ROS result in the death of cancer cells. Importantly, tumour cells utilize the cellular antioxidant system to counteract the pro‐death effects of ROS. Accumulating evidence shows that proteins with antioxidant activity are involved in tumorigenesis and metastasis.^38^, ^39^ However, the role of the antioxidant system in HCC carcinogenesis and metastasis is far from clear. Exploring the effects of SRXN1 and its regulation would greatly help in identifying novel roles of the antioxidant system in HCC carcinogenesis and metastasis.

SRXN1 has been reported to be associated with tumorigenesis by various cancers. The antioxidant function of SRXN1 was mediated by its effect on catalysing reduction of hyperoxidized 2‐Cys peroxiredoxins (Prxs). There are many studies describing the regulatory effect of SRXN1 on pivotal signalling pathways involved in tumorigenesis. Nancy H. Colburna's group reported that SRXN1 promotes progression of lung cancer by modulating phosphokinase signalling.^40^ That group also identified SRXN1 as a target of AP‐1 in skin malignancies.^41^ Additionally, SRXN1 was identified as a pro‐metastasis factor by enhancing EGFR signalling by Qiou Wei's group.^19^ Besides, SRXN1 expression is associated with survival outcome in breast cancer.^42^ It has been reported that SRXN1 promotes metastasis of cervical cancer by regulating the Wnt/β‐catenin signalling pathway.^18^ Although these results collectively indicate that SRXN1 is critically involved in carcinogenesis and metastasis of various cancers. The role of SRXN1 in HCC tumorigenesis has not been reported. Importantly, we identified BTG2 as a downstream target of SRXN1; This provides a novel layer of evidence for the tumour‐promoting function of SRXN1 and its regulatory network in HCC tumour metastasis.

In this study, we discovered that SRXN1 expression is up‐regulated in tumour tissue compared with adjacent nontumour tissue. Our results reveal a novel role for SRXN1 in HCC metastasis. p65 is a key member of NF‐κB signalling pathway. This pathway is considered to promote survival of cancer cells through modulating its downstream targets. Plenty of studies indicate that p65 was aberrantly up‐regulated in HCC cancer.^43^ ROS is also reported to be closely correlated with NF‐κB signalling.^44^ By conducting a series of experiments, we validate that modulating SRXN1 would influence ROS level and its effects on NF‐κB, then leading to metastasis of HCC. To screen possible downstream targets of SRXN1 modulating its biological function in promoting proliferation and metastasis, we carried out RNA‐seq experiments. We acquired a plethora of differential expressed genes in SRXN1 knockdown cells, of which BTG2 was up‐regulated and *P* value changes most (*P* = 2.045 × 10^−62^). These results led us to further investigate the role of BTG2 as a downstream target of SRXN1. BTG2 was previously shown to inhibit cell proliferation.^21^ Some studies have mentioned that BTG2 was associated with metastasis of malignant cancers,^45^, ^46^ but the detailed mechanisms are not clarified. In our work, we demonstrated that BTG2 suppressed EMT and metastasis in HCC tumours. SRXN1‐regulated ROS modulated p65, which inhibited BTG2 expression and ultimately led to migration and invasion by HCC cells, as well as tumour metastasis in vivo. Moreover, the relationship between BTG2 and EMT was first reported in our work. Importantly, SRXN1 inhibition increased expression of BTG2 indicated that up‐regulated SRXN1 might attenuate BTG2 expression, leading to increased proliferation of HCC cells. These findings will help the exploration of BTG2 as a potential tumour suppressor. However, how BTG2 inhibits EMT and promotes proliferation warrants further study. We are still investigating whether BTG2 directly interacts with molecules involved in EMT.

HCC often occurs on the background of chronic liver disease, and patients are often picked up at late stages. Findings from this study would help to exploit novel drugs targets in treating malignant and advanced HCC. Additionally, in this study, we clarified how SRXN1 modulated downstream signalling to regulate HCC tumorigenesis and metastasis. But how SRXN1 is regulated in HCC requires further clarification. There are no studies clarifying this regulation. SRXN1 could be exploited as a drug target to inhibit metastasis and recurrence in HCC patients. Furthermore, elucidation of the roles of SRXN1 in tumorigenesis and metastasis of HCC would help to identify the roles of SRXN1 in other malignant cancers. In conclusion, we unveiled a pro‐tumorigenic and pro‐metastasis of SRXN1 in HCC by regulating ROS/p65/BTG2 signalling. These results provide theoretical proof for exploiting SRXN1 as a novel target for treating HCC and other malignant cancers.

## CONFLICT OF INTEREST

The authors confirm that there are no conflicts of interest.

## AUTHOR CONTRIBUTION

**Xiufang Lv:** Investigation (equal). **Hailing Yu:** Investigation (equal). **Qianqian Zhang:** Formal analysis (equal). **Yongquan Huang:** Methodology (equal). **Xiaopeng Hong:** Resources (equal). **Ting Yu:** Resources (equal). **Huimin Lan:** Methodology (equal). **Chaoming Mei:** Methodology (equal). **Wenkai Zhang:** Software (equal). **Hui Luo:** Conceptualization (equal); Writing‐original draft (equal); Writing‐review & editing (equal). **Pengfei Pang:** Conceptualization (equal); Writing‐original draft (equal). **Hong Shan:** Funding acquisition (equal).

## Supporting information

Fig S1

## Data Availability

The data used in the current study are available from the corresponding author on reasonable request.
