# Supplementary figures and images for: SRXN1 stimulates hepatocellular carcinoma tumorigenesis and metastasis through modulating ROS/p65/BTG2 signalling
Source: J Cell Mol Med. 2020 Aug 3;24(18):10714–29. doi: 10.1111/jcmm.15693 (PMC7521256; doi:10.1111/jcmm.15693)

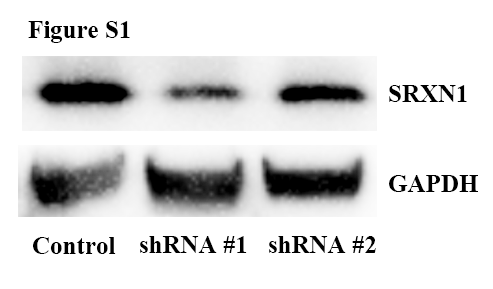

Supplement: Supplementary file 1 — Fig S1 [file JCMM-24-10714-s001.tif]
